# Robert J. Gorlin: Personal Memory of a Friend and Mentor in Clinical Genetics

**DOI:** 10.3390/audiolres13060081

**Published:** 2023-11-23

**Authors:** Bruno Dallapiccola, Rita Mingarelli

**Affiliations:** Ospedale Pediatrico Bambino Gesù, IRCCS, 00165 Rome, Italy; bruno.dallapiccola@opbg.net

Robert J. Gorlin (Bob), a pioneer in the fields of dentistry, surgical pathology, otolaryngology, dermatology and medicine, has been described as “a geneticist for all countries” [1]. No doubt, hundreds of physicians worldwide benefited either from his practice directly or by receiving advice for their patients. This also applies to the scientific community of Italian clinical geneticists. Many of us first became aware of Bob by reading his prolific production of scientific articles, books chapters and edited or coauthored books [2], including the monumental *Syndromes of the Head and Neck* [3] and *Hereditary Hearing Loss and its Syndromes* [4]. 

For half a century, Robert Gorlin was at the forefront of research in oral and maxillofacial pathology, craniofacial disorders, hereditary hearing loss, defects and syndromes, 46 of which he first recognized, making him one of the most quoted clinical geneticists, second only to Victor McKusick [5]. His career has crossed the pre-genomic era and has fully shown the power of dysmorphology in accompanying the progress of medical genetics. In this respect, he acted as one of the most representative messengers of James Paget, who reminded us that “not one [rare defect and feature] is without meaning, not one that might not become the beginning of excellent knowledge” [6].

During the very early 90s, we came in touch with Bob for the first time. At that time, we were appointed at the Scientific Directorate of the largest hospital in South Italy, Casa Sollievo della Sofferenza at San Giovanni Rotondo in the Apulia region, with the goal of implementing genetic research and translating it into clinical practice. As part of this project, since 1991, the School of Medical Genetics was raised within the hospital, which remained on training until 2009. Each year, about 200 students attended this school, which was considered the most authoritative clinical genetic academy in Italy at the time. The school’s schedule included lectures delivered by distinguished clinicians, dysmorphologists and geneticists, enrolled worldwide based on their significant contribution to particular diseases, and the discussion of puzzling patients often affected by undiagnosed disorders. In this context, we asked Bob to become a member of our faculty. He was genuinely enthusiastic for this invitation and, for three years, we had the honor of having him onboard and appreciating his stimulating and enjoyable talks, the pleasure of dealing with young colleagues, clinicians and scientists, the ability to listen and be a thoughtful analyst, humility and friendship. Alongside being an excellent observer, he was aided by his unusual ability to retain what he had seen or read in outlining a specific diagnosis, and personal observations or information submitted by colleagues to lump published data. 

In those years, Bob became more and more popular among the Italian human geneticists. In 1997, he was the guest and keynote speaker at the 12th Convention of the Italian Association of Medical Genetics in Spoleto, where he was awarded with the Phoenix Anni Verdi Prize in recognition of his outstanding career (Figure 1). 

We met and interacted with Bob in other contexts, such as the Gorlin Conferences on Dysmorphology in Minneapolis, or requesting his suggestion and insight in solving some of our undiagnosed patients. The success rate in these efforts was quite high, as expected from his unique clinical skills.

Our last personal memories of Bob date back to his illness, which he lived through with lucidity and great courage. In the early stage of the disease, he did not lose his good humor and optimism, telling us some anecdotes about the therapeutic sessions he shared with a similarly affected famous king. However, one day, we became fully aware of the decline of his health, when he sent us his last heartfelt and sad message: “I regret not being able to be with you once again, to share these days of culture and culinary pleasure, but now I’m on a boat that is moving away from the shore. I see you on the port, and leave you for other shores, with the memory of every moment spent together”.

Several years after that message, we keep the remembrance of a great, humble, curious, enthusiastic master of medicine, who has generously shared his knowledge and unique experience with thousands of people, including students, colleagues and patients. It was a huge honor and an enormous privilege for us to have met him, to have been among these people and to have traveled a piece of our professional career together.

## Figures and Tables

**Figure 1:**
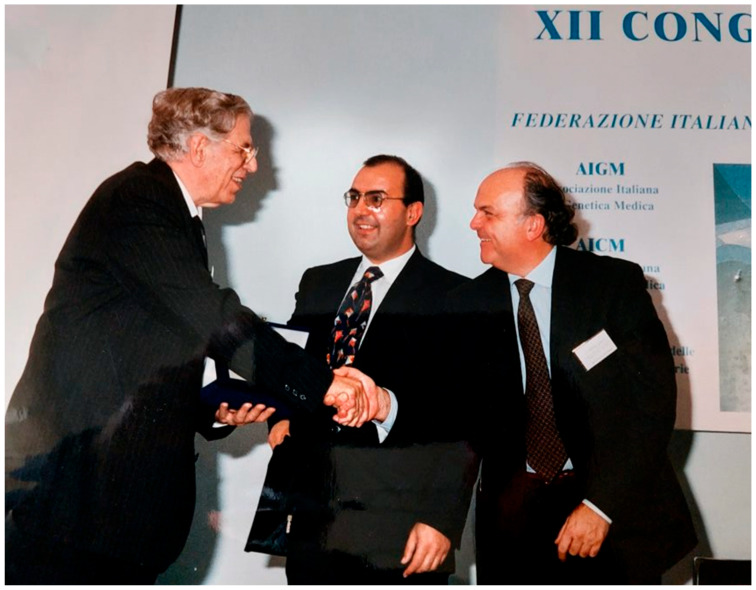
Robert Gorlin (left) awarded by Bruno Dallapiccola (right) in 1997 with the Phoenix Anni Verdi prize.
